# A good response to furmonertinib fourth-line treatment of an advanced lung adenocarcinoma patient with EGFR exon20in and PIK3CA mutation: a case report and literature review

**DOI:** 10.3389/fonc.2024.1467722

**Published:** 2024-12-17

**Authors:** Kai Sun, Peng Wang

**Affiliations:** Department Oncology, Yidu Central Hospital of Weifang, Weifang, China

**Keywords:** EGFR-TKI, PIK3CA mutant, furmonertinib, synchronous cancer, EGFR-TKI acquired resistance

## Abstract

**Background:**

Lung cancer, including small cell lung cancer (SCLC) and non-small cell lung cancer (NSCLC), is the most prevalent cancer globally and remains the leading cause of cancer-related mortality. Epidermal growth factor receptor (EGFR) mutations, frequently observed in female NSCLC patients, have revolutionized treatment strategies with the advent of tyrosine kinase inhibitors (TKIs). These therapies significantly improve survival and are considered the standard of care for patients harboring EGFR mutations. However, most patients eventually develop resistance to EGFR-TKIs, leading to disease progression. Resistance mechanisms are classified as either EGFR-dependent or EGFR-independent, the latter involving bypass pathway activation, including dysregulation of downstream signaling cascades. EGFR-independent resistance often renders all EGFR-TKIs ineffective, necessitating further investigation into resistance mechanisms.

**Case summary:**

We report the case of a 63-year-old Chinese woman diagnosed with synchronous lung adenocarcinoma harboring an EGFR exon 21 far-loop insertion mutation and clear cell renal cell carcinoma (ccRCC). A multidisciplinary team recommended systemic therapy for the lung adenocarcinoma and clinical observation for ccRCC. First-line treatment with bevacizumab plus pemetrexed-carboplatin achieved a progression-free survival (PFS) of 7 months. Second-line treatment with sintilimab and nedaplatin resulted in a PFS of 4.9 months. Third-line therapy with sintilimab and anlotinib proved ineffective. In the fourth line, the patient received furmonertinib, a third-generation EGFR-TKI, based on the FAVOUR trial. This treatment achieved durable disease control with excellent tolerability, yielding a PFS of 27 months and ongoing clinical benefit.

**Conclusion:**

This case demonstrates that furmonertinib can provide significant clinical benefit to NSCLC patients with complex resistance mechanisms, including those involving the PIK3CA/mTOR pathway. These findings support its potential to overcome EGFR-TKI resistance and warrant further investigation in similar clinical contexts.

## Introduction

The epidermal growth factor receptor (EGFR) plays a pivotal role in regulating cellular processes such as proliferation, differentiation, division, and survival, and is intricately linked to the development of cancer (1). Recognized as a key therapeutic target in oncology, EGFR is frequently mutated in non-small-cell lung cancer (NSCLC) (2, 3). EGFR-tyrosine kinase inhibitors (TKIs) have demonstrated significant efficacy in eliciting tumor responses, particularly in NSCLC patients with EGFR mutations, surpassing traditional cytotoxic chemotherapy regimens.

Despite the efficacy of EGFR-TKIs, emerging evidence suggests that patients with advanced NSCLC and EGFR Exon 20 insertions (Exon 20ins) exhibit significant resistance to these inhibitors (4). Studies conducted across Asia, encompassing populations from China, Taiwan, and India, have consistently reported that metastatic NSCLC patients with EGFR Exon 20ins mutations show the worst progression-free survival (PFS) and overall survival (OS) when treated with first-generation EGFR TKIs as either first-line or subsequent therapy (5, 6). Structural analyses have implicated mutations in the EGFR drug-binding pocket, which may reduce the binding affinity of TKIs to the receptor.

Among the available third-generation EGFR-TKIs, osimertinib has emerged as a potential countermeasure, demonstrating its ability to overcome the reduced sensitivity to EGFR-TKIs observed in certain EGFR exon 20ins variants, both *in vitro* and *in vivo*. Nevertheless, the challenge of resistance to EGFR TKI therapy in EGFR exon 20ins remains formidable.

Furmonertinib, a novel third-generation EGFR-TKI, has shown promise in overcoming drug resistance mediated by the ATP-binding cassette transporters ABCB1 and ABCG2, which are central to the development of multidrug resistance in cancer patients receiving conventional chemotherapy. The mechanism of action of furmonertinib was characterized through ATPase assays, revealing its interaction with ABCB1 and ABCG2,suggesting a potential strategy to overcome resistance in EGFR exon 20ins-mutated cancers.

We herein report a case of the use of furmonertinib, to treat lung cancer with EGFR exon 20ins. Furmonertinib was effective in treating lung cancer as subsequent therapy even the exist of PIK3CA mutant.

## Chief complaints

A 63-year-old Chinese woman was referred to our hospital presenting with a cough and right-sided lumbar pain lasting for four months.

### History of present illness

The patient presented with cough, expectoration, and dull pain in the right side of the waist for more than four months. Symptomatic treatment outside the hospital was ineffective.

### History of past illness

The patient was previously healthy, with no significant history of illness, trauma, or surgery.

### Personal and family history

The patient’s personal and family medical histories were unremarkable, with no familial history of cancer and generally good health.

### Physical examination

No palpable enlargement of the superficial lymph nodes was observed. No dry or moist rales were heard on auscultation of both lungs. The heart rhythm was regular, with no murmurs auscultated. The abdomen was soft, non-tender, with no rebound tenderness or palpable masses. Additionally, no percussion tenderness was noted over the liver and kidneys.

### Imaging examinations

**Figure 1:**
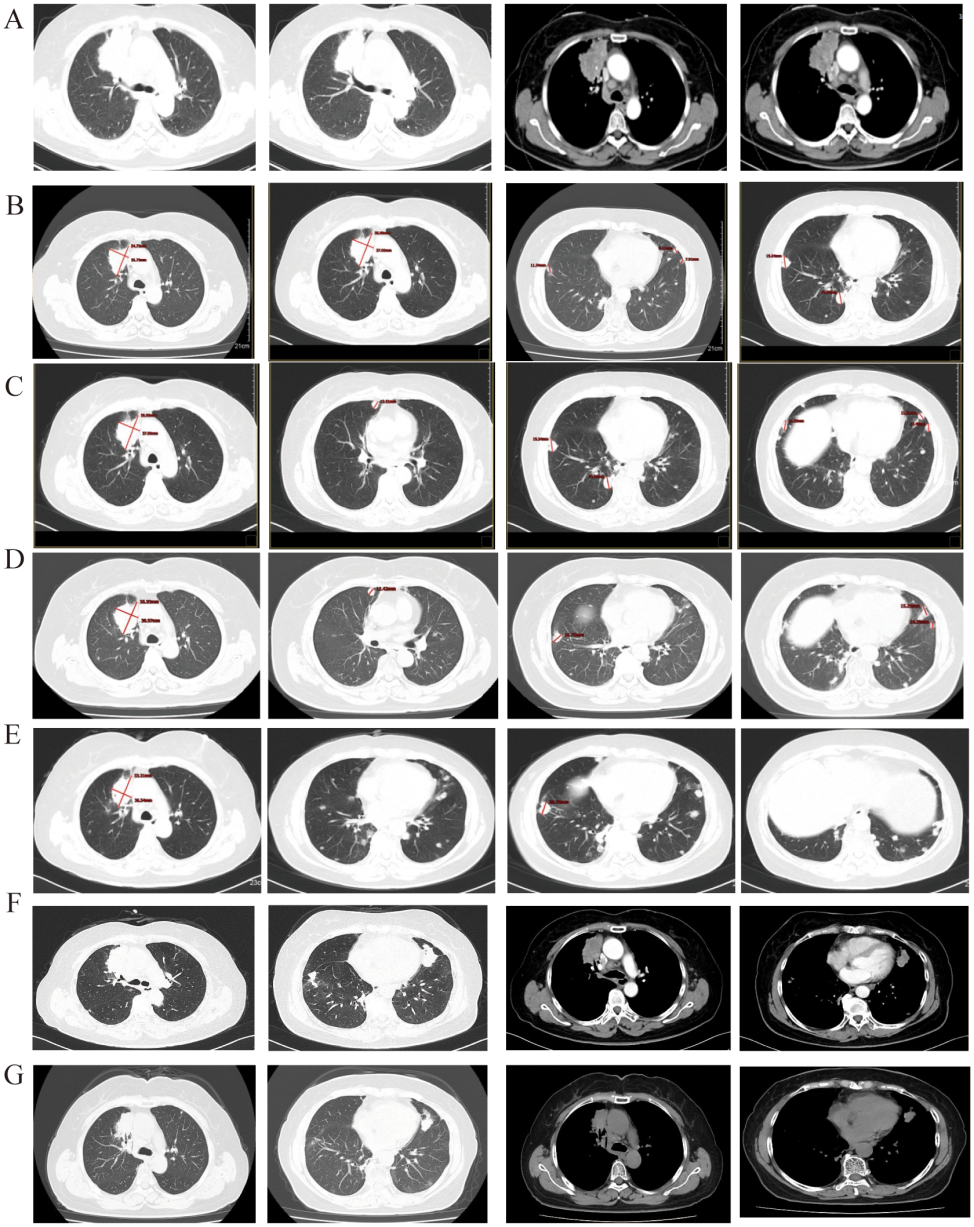
**(A)** Before treatment, the longest diameter of the target lesion is 6.3 cm + 1.6 cm to lung adenocarcinoma; **(B)** After 2 cycle of treatment, the primary lesion in the right lung has reduced compared to before, the efficacy evaluation was SD. **(C)** The size of the primary lung lesion shows no significant change compared to before, with multiple nodules of varying sizes seen in both lungs, some of which have increased in size compared to the previous observation. The efficacy evaluation was PD. **(D)** The primary lung lesion shows no significant change compared to before, while the metastatic lesions in both lungs have increased and enlarged compared to before. The efficacy evaluation was PD. **(E)** The primary lung lesion and the metastatic lesions in both lungs show no significant change compared to before and there is a trend of reduction. **(F)** The primary lung lesion and the metastatic lesions in both lungs show significant progression compared to before. **(G)** After 2 cycle threefold dosage furmonertinib treatment, the primary lung lesion and the metastatic lesions in both lungs show significant reduce.

Contrast-enhanced chest CT(CCT) revealed clear lung fields and a prominent mass with significant contrast enhancement in the upper lobe of the right lung, near the mediastinum. The lesion was 6.3 cm in its longest diameter and showed features typical of cancer, including pleural indentation, lobulation, and spiculation. Numerous small metastatic nodes were seen in both lungs, suggesting widespread metastasis. Additionally, multiple enlarged lymph nodes were identified in the mediastinum (**Figure 1A**). Unexpectedly, a distinct lesion was also detected in the right kidney. Neck ultrasound identified further swollen lymph nodes. The patient then underwent an enhanced MRI to exclude additional brain tumors.

### Pathology examinations

**Figure 2:**
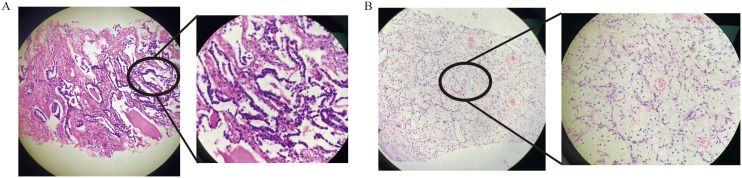
HE staining of lung adenocarcinoma diagnosis **(A)** and HE staining of renal clear cell carcinoma diagnosis **(B)**.

The patient may have synchronous malignancies, as it is extremely rare for lung cancer to metastasize from kidney cancer or vice versa. Both tumors were identified through needle biopsies. Histopathological and immunohistochemical analyses confirmed lung adenocarcinoma (LUAD) (**Figure 2A**) and clear cell renal carcinoma (ccRCC) (**Figure 2B**). Genome sequencing of the LUAD tissue identified two significant genetic mutations: an EGFR exon20ins and a PIK3CA H1047R mutation, with allele fractions of 28.85% and 18.66%, respectively (**Table 1**). According to the Fuhrman classification, the ccRCC is classified as Grade 1, indicating a low risk of progression and well-differentiated features.

**Table 1:** The patient's first genome sequencing results.

Gene	Mutation	Fraction
EGFR	exon20 c2303_2311dup p.S768_D770dup	28.85%
KRAS	No significant mutation	/
ALK	No significant mutation	/
ROS1	No significant mutation	/
MET	No significant mutation	/
BRAF	No significant mutation	/
FGFR1	No significant mutation	/
FGFR2	No significant mutation	/
HER2	No significant mutation	/
NRAS	No significant mutation	/
HRAS	No significant mutation	/
RET	No significant mutation	/
TSC1	No significant mutation	/
AKT1	No significant mutation	/
PIK3CA	exon21 c3140A>G p.H1047R	18.66%
NTRK1	No significant mutation	/
NTRK2	No significant mutation	/
NTRK3	No significant mutation	/

## Final diagnosis

Based on pathological, imaging, and laboratory findings, both LUAD and ccRCC were diagnosed. “LUAD was staged as IVa (cT4N3M1a) with EGFR and PIK3CA mutations, according to the eighth edition of the TNM staging system. Similarly, ccRCC was staged as I (cT1bN0M0), also according to the eighth edition of the TNM staging system, and classified as Grade 1 by Fuhrman classification.

### Treatment and follow Up

#### First-line

According to the 2020 CSCO guidelines for non-small-cell lung cancer (7), the first-line treatment for LUAD with EGFR exon 20 mutation is chemotherapy plus bevacizumab. Similarly, the CSCO guidelines for kidney cancer recommend bevacizumab plus IFNα-2b for ccRCC. Due to overlapping treatment protocols, the patient received combined therapy with bevacizumab and pemetrexed-carboplatin. After two cycles of combined therapy, the patient’s condition was assessed as stable disease (SD) according to RECIST 1.1 criteria, with a 21.5% reduction in the maximum diameter of the lung’s target lesion and significant shrinkage of non-target lesions (**Figure 1B**). The ccRCC also remained SD. After six cycles of combined treatment and subsequent maintenance therapy with bevacizumab and pemetrexed, the LUAD progressed locally (**Figure 1C**), while the ccRCC continued to exhibit sustained SD. The PFS from the first-line treatment was 7 months.

#### Second-line

The patient received sintilimab, a PD-L1 inhibitor, combined with nedaplatin and paclitaxel as a second-line treatment. After the first four cycles, she showed SD for 4.9 months, with a slight reduction in the maximum diameter of the target lesion. Following six cycles of treatment, the disease locally advanced again (**Figure 1D**). However, the ccRCC remained stable. The PFS for this second-line treatment was 4.9 months.

#### Third-line

According to the 2021 CSCO guidelines for NSCLC treatment (8), the patient received sintilimab combined with anlotinib, an anti-angiogenic agent. The third-line treatment was ineffective, as the disease rapidly progressed due to new lung metastases(**Figure 1E**). However, the ccRCC remained stable.

#### Fourth-line

**Figure 3:**
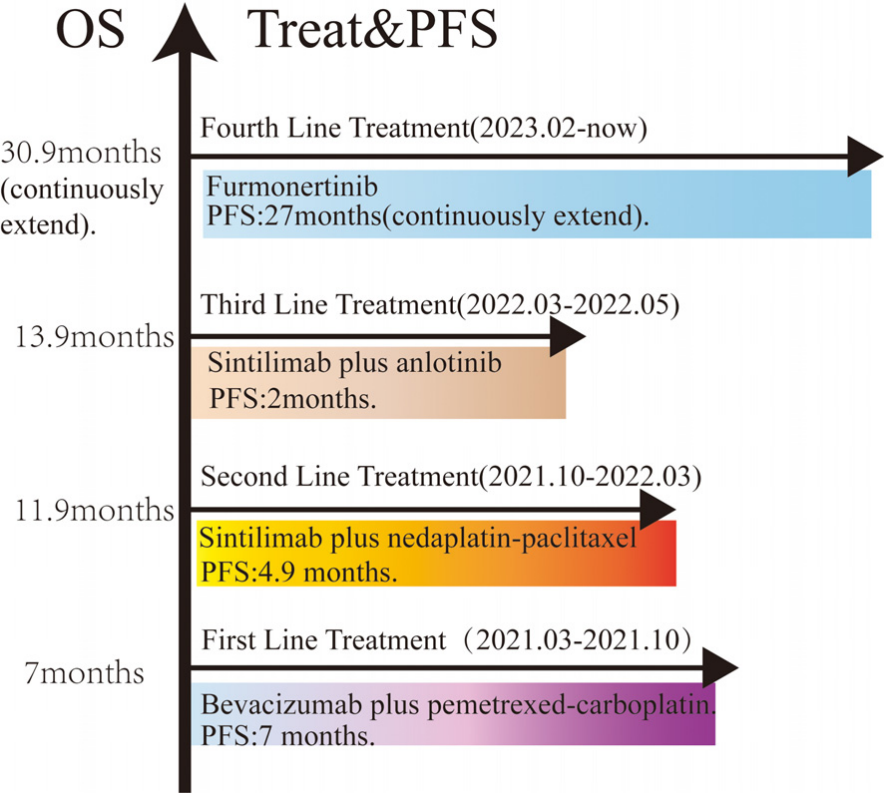
An overall review of the efficacy evaluation and treatment duration for this patient.

The patient commenced furmonertinib, an EGFR-mutant targeted therapy, as fourth-line treatment following the publication of the FAVOUR study. This drug has provided sustained benefits. After two months of treatment with furmonertinib, the disease achieved a partial response (PR). The ccRCC remained stable. The PFS for the fourth-line treatment has reached 24 months. Recently, the disease was progressed again due to locally advancement (**Figure 1F**). The patient underwent genome sequencing again. The second genome sequencing result was similar to the first time: an EGFR exon20ins and a PIK3CA H1047R mutation, with allele fractions of 51.07% and 13.66%, respectively (**Table 2**). Due to prolonged cancer control and the recurrence of the same gene mutation, the patient was administered a threefold dose of furmonertinib (240mg QD).After two cycles treatment, the patient underwent another CCT to assess treatment efficacy. The lung lesion had significantly reduced compared to prior scans (**Figure 1G**). The patient has achieved a total of 27 months of PFS with furmonertinib, and the PFS continues to extend (**Figure 3**).

**Table 2:** The patient's second genome sequencing results.

Gene	Mutation	Fraction
EGFR	exon20 c2303_2311dup p.S768_D770dup	51.07%
KRAS	No significant mutation	/
ALK	No significant mutation	/
ROS1	No significant mutation	/
MET	No significant mutation	/
BRAF	No significant mutation	/
FGFR1	No significant mutation	/
FGFR2	No significant mutation	/
HER2	No significant mutation	/
NRAS	No significant mutation	/
HRAS	No significant mutation	/
RET	No significant mutation	/
TSC1	No significant mutation	/
AKT1	No significant mutation	/
PIK3CA	exon21 c3140A>G p.H1047R	13.66%
NTRK1	No significant mutation	/
NTRK2	No significant mutation	/
NTRK3	No significant mutation	/

The patient tolerated the dose escalation well, with manageable side effects. During high-dose therapy, the primary side effect was Grade 1 oral mucositis classified as per the Common Terminology Criteria for Adverse Events (CTCAE) version 5.0. The patient reported mild oral pain, occurring occasionally while chewing, which was tolerable and did not significantly impact daily activities or quality of life. No medical intervention was required, and the treatment dose of furmonertinib was maintained without adjustment.

## Discussion

Lung cancer, classified into small cell lung cancer (SCLC) and Non-small cell lung cancer (NSCLC), is the most prevalent cancer globally and is the predominant contributor to mortality attributed to cancer (9, 10).

EGFR mutations are commonly observed in most female NSCLC patients. The use of TKIs in patients who harbor EGFR mutations significantly improves overall survival (11–13).

### Developing treatment plan

This patient has synchronous primary cancers: LUAD and ccRCC. According to the Fuhrman classification, this patient is classified within the low-risk subgroup for ccRCC, which is likely to remain stable even without treatment. Therefore, clinical observation can be a viable option for managing low-risk ccRCC. However, LUAD requires timely treatment. Given the patient’s synchronous cancers, treatment decisions should require a multidisciplinary team approach to address both conditions effectively. The 2020 CSCO treatment guidelines for NSCLC (7) recommend bevacizumab combined with chemotherapy for LUAD with an EGFR mutation, which can also provide therapeutic benefits for ccRCC. A real-world study (14) demonstrated that chemotherapy is more effective than EGFR-TKIs for LUAD with an EGFR exon20ins. This is because the mutation alters the conformation at the kinase active site, reducing the efficacy of early-generation EGFR-TKIs. Combining chemotherapy with bevacizumab has been shown to improve overall survival, and this approach resulted in a PFS of 7 months. The second-line treatment, based on the Orient-11 study (15), combines immunotherapy with chemotherapy, providing a longer PFS for non-squamous cell carcinoma NSCLC patients. This plan resulted in a 4.9-month PFS. As the cancer advanced, the efficacy of anti-cancer therapies diminished. The third-line treatment involved anti-angiogenic therapy, a standard choice for subsequent treatment in LUAD, which sometimes leads to favorable clinical outcomes. Due to drug marketing policies, the patient received sintilimab for free, combining anti-angiogenic therapy with immunotherapy. Despite this combination, no significant PFS improvement was observed. When the LUAD advanced again, the FAVOUR study was published, indicating that patients with an Exon 20 insertion could benefit from furmonertinib, which demonstrates a favorable safety profile. According to the study, the patient achieved a long-term PFS.

After 24 months of treatment with furmonertinib at a standard dose of 80 mg daily, the patient experienced disease progression, as evidenced by an increase in the size of the pulmonary lesion. The second genome sequencing revealed that the EGFR exon20ins mutation persisted and demonstrated an increased mutant allele frequency compared to baseline, without the emergence of any new resistance-associated genetic alterations. This finding suggested that the resistance mechanism remained EGFR-dependent, likely due to increased mutant EGFR burden, which rendered the standard dose insufficient to suppress tumor progression effectively. In response, the treatment regimen was adjusted to a higher dose of furmonertinib at 240 mg daily to enhance EGFR inhibition. After two cycles of high-dose furmonertinib, a follow-up imaging assessment demonstrated a significant reduction in the size of the pulmonary lesion, suggesting effective disease control.

### EGFR exon20 insertion mutation in NSCLC

**Figure 4:**
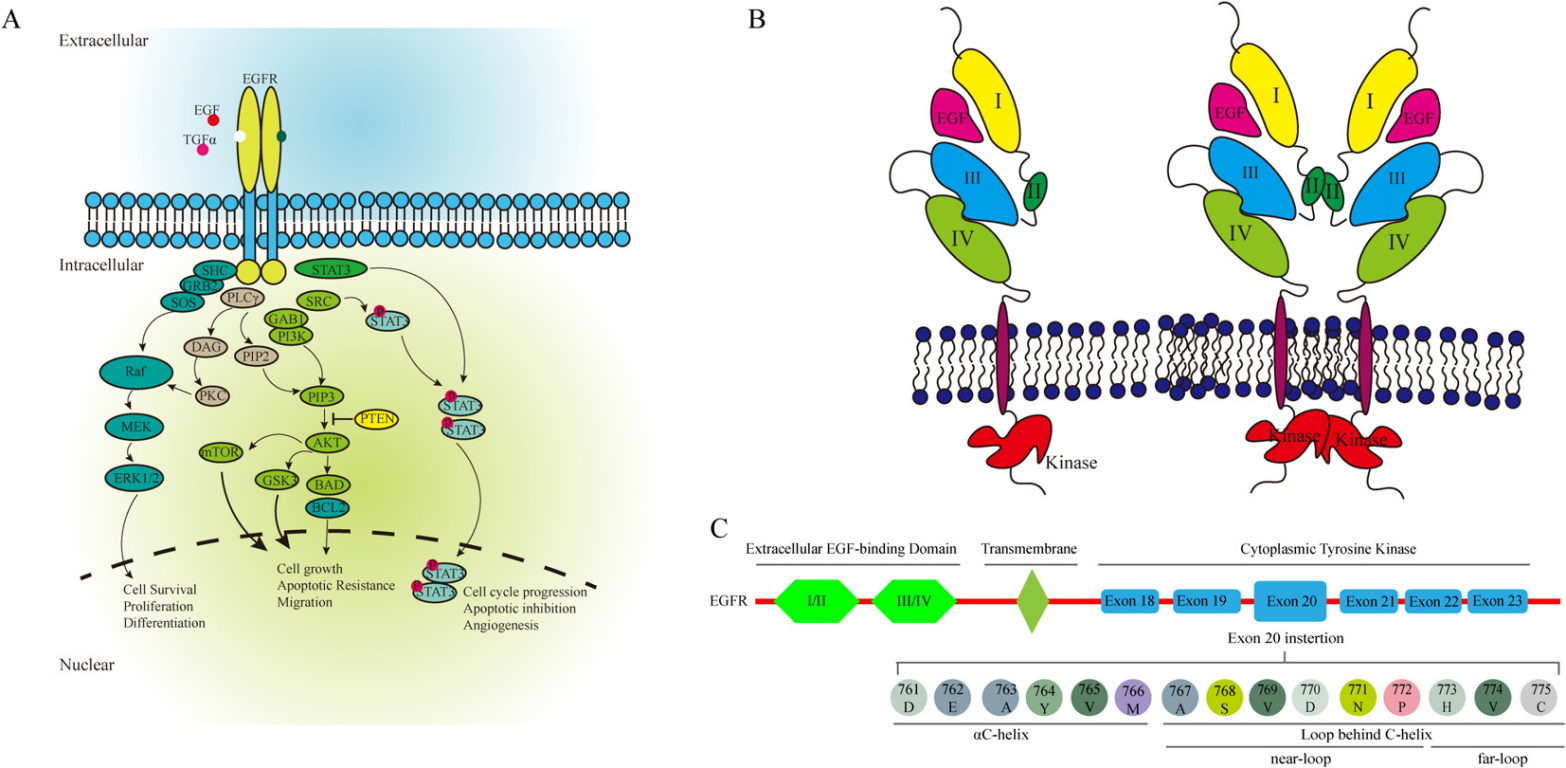
The EGFR associated pathway **(A)**, the activation of EGFR **(B)**, and the structure of EGFR **(C)**.

EGFR is a glycoprotein composed of three principal domains: an extracellular EGF-binding domain, a transmembrane region and a cytoplasmic tyrosine kinase domain essential for regulating catalytic activity. The cytoplasmic domain includes a smaller N-terminal lobe and a larger C-terminal lobe, separated by the ATP-binding cleft. Ligand binding to EGFR triggers dimerization, activating the kinase domain and initiating downstream signaling pathways (**Figure 4A**). Common EGFR mutations in NSCLC include exon 19 deletions (exon19del) and the L858R substitution in exon 21, together accounting for 85% of EGFR mutations observed in NSCLC (11, 16, 17).

EGFR exon20ins account for approximately 4% to 12% of all EGFR mutations, making them the third most frequent (18, 19). These mutations are predominantly found in women, non-smokers, and Asians (10, 18). EGFR ex20ins mutations occur primarily in the C-terminal loop of the αC-helix (ex20ins-L) and within the αC-helix itself, and can be further classified into near-loop (AA767–772) and far-loop (AA773–775) subtypes. Far-loop mutations are notably resistant to first- and second-generation EGFR-TKIs. The effectiveness of EGFR-TKI is always insufficient in the patients with EGFR exon21 far-loop insertion (14, 20) (**Figure 4B**).

Osimertinib, a third-generation EGFR-TKI, exhibits limited efficacy in NSCLC patients with EGFR exon20ins. Studies reveal low ORR (5% to 6.5%) and short mPFS (2.3 to 3.6 months). A phase II trial doubling the recommended dose of osimertinib in such patients did not reach 30% ORR, recording a 24% ORR and a median PFS of 9.6 months (21). Hence, osimertinib may offer only modest benefits at higher doses.

Furmonertinib has shown effectiveness as a first-line treatment in advanced NSCLC patients with EGFR exon20ins, achieving mPFS of 8.13 to 10.90 months (22). It and its main metabolite effectively target cancers with sensitive EGFR mutations and the T790M resistance mutation, while minimally affecting wild-type cells. The FURLONG study demonstrated that furmonertinib significantly prolongs mPFS compared to gefitinib in Chinese patients with EGFR mutation-positive advanced NSCLC (22). Ongoing clinical trials also report favorable outcomes and tolerability for furmonertinib in patients with these specific mutations.

### Novel Ex20ins inhibitors

Recent advancements have led to the development of novel targeted therapies for NSCLC patients with EGFR ex20ins. Drugs such as mobocertinib, amivantamab, CLN-081, and sunvozertinib have demonstrated promising therapeutic effects in clinical trials (23). Mobocertinib, a small molecule EGFR/HER2 TKI, is designed to specifically target EGFR exon20ins (18, 24, 25). It achieves enhanced selectivity by irreversibly binding to the cysteine-797 (c-797) residue of EGFR, with clinical studies reporting an investigator-confirmed response rate of 43% and a median PFS of 7.3 months in NSCLC patients. Amivantamab (26–29), an innovative bispecific monoclonal antibody targeting EGFR and c-MET, has shown antitumor efficacy through multiple mechanisms, including disruption of ligand binding and receptor phosphorylation, and immune cells engagement. In the CHRYSALIS phase I clinical trial, previously treated NSCLC patients with ex20ins mutations exhibited an ORR of 40% and a median PFS of 8.3 months (26). CLN-081, an oral irreversible EGFR-TKI, selectively targets exon20ins mutations and has demonstrated efficacy in preclinical studies by inhibiting various exon 20 insertion mutations. Clinical trials of CLN-081 have shown a PR rate of 40% and stable disease in 56% of NSCLC patients with ex20ins mutations. Sunvozertinib, a selective small molecule inhibitor of EGFR exon20ins, has exhibited remarkable antitumor activity in a pivotal study, with a confirmed ORR of 60.8% among Chinese NSCLC patients harboring these mutations (30, 31).

### EGFR-dependent mechanisms of resistance to third-generation EGFR-TKIs

Acquired resistance to third-generation EGFR-TKIs represents a significant clinical challenge in the treatment of EGFR-mutant lung adenocarcinoma. Among EGFR-dependent resistance mechanisms, secondary mutations within the EGFR kinase domain are predominant. The C797S mutation, which disrupts the covalent binding of third-generation EGFR-TKIs, is the most frequently observed, often typically emerging following prolonged therapy (32). Additional mutations, including T790M, L792H, G796R, M766Q, and L798I, can modify the kinase domain and reduce drug efficacy (33–36). Furthermore, the formation of EGFR heterodimers, such as EGFR-HER2 or EGFR-HER3, initiates compensatory signaling pathways that bypass EGFR inhibition. This dimerization drives oncogenic downstream signaling, thereby sustaining tumor cell proliferation and survival. An additional key mechanism is the activation of the PKCδ signaling pathway, which leads to the nuclear translocation of PKCδ and subsequent activation of AKT and NF-κB signaling, promoting cell survival and therapeutic resistance. Together, these mechanisms highlight the complexity of EGFR-dependent resistance and underscore the need for therapeutic strategies that target both the EGFR kinase domain and its downstream effectors. Combination therapies, integrating EGFR inhibitors with agents targeting HER2/3 or the PKCδ pathway, may represent a promising approach to overcoming resistance. Further investigations are warranted to develop strategies that prevent or delay the emergence of these resistance mechanisms, ultimately improving outcomes for patients with EGFR-mutant lung adenocarcinoma.

### PIK3CA mutation in NSCLC

The PIK3CA gene encodes the alpha isoform of the catalytic subunit of phosphatidylinositol 3-kinase (PI3K) and plays a key role in the activation of the PI3K/AKT/mTOR signaling pathway, which is critical for regulating cancer-associated cellular processes (37). PIK3CA mutations occur in various cancers, at frequencies of 5% to 8% in NSCLC cases and have been identified in approximately 6.33% of Chinese pan-cancer samples. Key mutation hotspots include E545K/Q/A/V/D/G, E542K, and H1047R/L/Y. These mutations promote cellular survival and proliferation by activating the PI3K/AKT/mTOR pathway. Notably, the H1047R mutation, one of the most common, is located in the kinase domain and plays a significant role in promoting cell growth and survival. This mutation also contributes to resistance to EGFR-TKIs in lung cancer by activating downstream effectors such as AKT and mTOR (38). Furthermore, studies suggest that NSCLC patients with concurrent EGFR and PIK3CA mutations experience significantly shorter progression times and reduced overall survival when treated with EGFR-TKI therapy, compared to those with only EGFR mutations (17, 38).

### PIK3CA inhibitors in cancer

In recent years, many PI3K/AKT pathway specific TKIs were developed, such as pan-AKT inhibitors, dual PI3K/mTOR inhibitor,PI3K subtype inhibitor and mTOR inhibitors. Alpelisib, a selective PI3Kα inhibitor, has demonstrated a notable efficacy in targeting PIK3CA-mutated tumors, a significant genetic subgroup within breast cancer. The phase III SOLAR-1 trial provided evidence that the therapeutic synergy of alpelisib with fulvestrant in endocrine therapy significantly enhanced PFS in patients with PIK3CA-mutated, ER+ metastatic breast cancer who had previously undergone antiestrogen treatment (39). Alpelisib emerges as a beacon of promise as a PI3Kα-specific inhibitor. Taselisib, a PI3Kα-specific inhibitor, has been scrutinized in clinical trials for its potential role in breast cancer treatment (40, 41). The phase III SANDPIPER trial demonstrated a modest, but statistically significant, enhancement in PFS with the taselisib and fulvestrant combination, as opposed to fulvestrant monotherapy, in ER+ advanced breast cancer patients who had encountered progression during or subsequent to aromatase inhibitor (AI) therapy (41). This improvement, though not substantial, was notable, with a median PFS of 7.4 months versus 5.4 months (p=0.0037), irrespective of the PIK3CA mutation status. Conversely, the phase II LORELEI trial did not reveal a significant divergence in pathologic complete response (pCR) rates between the taselisib and letrozole combination and letrozole alone in the neoadjuvant treatment of early-stage, ER+/HER2- breast cancer patients, whether they harbored PIK3CA mutations or not (40). Both the SANDPIPER and LORELEI trials reported high incidences of severe adverse effects associated with taselisib treatment, resulting in a significant rate of treatment discontinuation—17% and 11%, respectively. These safety concerns have overshadowed the drug’s potential benefits and have impeded its progression in clinical development.

This patient was tested out two gene mutants, EGFR exon20ins and PIK3CA H1047R. As a previous study, EGFR-TKIs might not benefit this patient. But furmonertinib resulted in a long PFS, even as fourth line treatment. The reasons of EGFR-TKIs working in this patient are complicated. This patients was subjected with a history of multi-line therapy, including chemotherapy, immunotherapy and anti-angiogenic therapy. Polytherapy is one of the key points for this patient. It might change the gene expression profile, the EGFR mutant might be more in allele fraction, or the PIK3CA/AKT/mTOR pathway might not be primary pathway in cancer cell proliferation for this patient. Another reason might be the drug, furmonertinib. Furmonertinib could irreversibly inhibits EGFR with resistance (T790M mutation) or activating mutations. Previous study demonstrated that furmonertinib may be suitable as a first-line treatment option for patients with EGFR exon20 ins, as it can significantly improve symptoms and prolong survival, with fewer and manageable side effects.

This case study has several limitations. When diagnosing LUAD and ccRCC, additional examinations, such as bone scans and positron emission tomography-computed tomography (PET/CT), should be completed. The genetic status of the thoracic lesions must be confirmed by comprehensive Gene Testing Methods, such as Whole-Genome Sequencing (WGS) or Transcriptome Sequencing (RNA-Seq), to test for both known and unknown genetic mutations. Regarding this patient, three critical questions need addressing: What is the next treatment plan after the fourth progression? Is PD-L1 detection necessary in lung tissue? When is it appropriate to operate on ccRCC? Our research group is committed to augmenting the scope of our study by broadening the sample cohort, thereby facilitating a more definitive assessment of the clinical efficacy of furmonertinib as an effective therapeutic treatment for individuals afflicted with NSCLC that exhibit EGFR exon20 ins and other gene mutant. We will also explore the potential clinical benefit by which furmonertinib has effects against NSCLC with EGFR ex20ins mutation combined other treatment.

## Data Availability

The raw data supporting the conclusions of this article will be made available by the authors, without undue reservation.
